# Recovery and long-term health outcomes of SARS-CoV-2 infection in a prospective cohort in an urban setting, Kenya

**DOI:** 10.1080/16549716.2025.2500795

**Published:** 2025-05-30

**Authors:** Isaac Kisiangani, Ângela Jornada Ben, Elke Wynberg, Welcome Wami, Samuel Iddi, Idah Kinya, Anna Vassall, Catherine Kyobutungi, Abdhalah Ziraba, John Njeru, Olive Mugenda, Marion Wangui Kiguoya, Mutambuki Kimondo, Geoffrey Githua, Menno D. de Jong, Shukri F. Mohamed, Gershim Asiki, Constance Schultsz

**Affiliations:** a Health & Wellbeing, African Population and Health Research Center, Nairobi, Kenya; b Department of Global Health, Amsterdam Institute for Global Health and Development, Amsterdam UMC, location University of Amsterdam, Amsterdam, The Netherlands; c Amsterdam Institute for Global Health and Development, Amsterdam, the Netherlands; d Department of Health Sciences, Faculty of Science, Vrije Universiteit Amsterdam, Amsterdam Public Health research institute, Amsterdam, The Netherlands; e Mahidol-Oxford Tropical Medicine Research Unit (MORU), Faculty of Tropical Medicine, Mahidol University, Bangkok, Thailand; f Research & Clinical Trials, Kenyatta University Teaching, Referral and Research Hospital, Nairobi, Kenya; g Department of Global Health, London School of Hygiene and Tropical Medicine, London, UK; h Centre for Microbiology Research, Kenya Medical Research Institute (KEMRI), Nairobi, Kenya; i Department of Medical Microbiology & Infection Prevention, Amsterdam UMC, location University of Amsterdam, Amsterdam, The Netherlands

**Keywords:** Long COVID, fatigue, post-acute COVID-19 syndrome, symptoms, risk factors

## Abstract

**Background:**

Evidence on long COVID remains limited in sub-Saharan countries.

**Objective:**

This study explored the occurrence of COVID-19-related symptoms and factors affecting recovery and long COVID severity in Nairobi, Kenya.

**Methods:**

A prospective cohort of individuals testing positive for SARS-CoV-2 between February 2022 and February 2023 was followed until June 2023. COVID-19-related symptoms were assessed every three months. Time to recovery was analyzed using survival analysis, while factors affecting recovery factors and long COVID severity using Cox proportional hazard and Poisson regression, respectively.

**Results:**

Among 291 participants (median age 34, 59.1% female), 42 (14%) had severe/critical infection. At 6 and 12 months post-positive PCR, 53.1% and 33.5% had ≥ 1 COVID-19-related symptoms, respectively. Fatigue (40.2%), pain (36.8%), sore throat (36.8%), headaches (36.4%), and loss of strength (31.6%) were most common. Median time to recovery was longer for severe/critical cases than mild/moderate (234 vs 206 days, *p* = 0.016). Participants aged 40–64 years experienced slower recovery than those aged < 40 years (aHR = 0.635 [95%CI, 0.429;0.941]). Participants with tertiary education recovered faster than those with primary education (aHR = 1.869 [95%CI, 1.050;3.327]). Long COVID severity was associated with female sex (aIRR = 1.418 [95%CI; 1.078;1.864]), tertiary education (aIRR, 0.489 [95%CI, 0.415;0.576]), and ≥ 1 comorbidity (aIRR = 2.415 [95%CI, 1.639;3.559]).

**Conclusions:**

Six months post-infection, half had lingering symptoms, with a third still affected after a year. Recovery was faster in younger, educated individuals, while severe long COVID was more common in women, those with low education and pre-existing conditions. The burden of long COVID in Kenya requires support for vulnerable groups.

## Paper context

**Main findings**Six months after infection, half of the participants still had COVID-19-related symptoms, and a third continued to experience them after a year, with fatigue being the most common symptom. Faster recovery time from symptoms was associated with younger age and higher education level. Severe Long COVID was more likely in women and individuals with lower levels of education or pre-existing health conditions. **Added knowledge** The study provides a systematically tracking of Long COVID symptoms in a Kenyan cohort over 12 months, as well as epidemiological data on occurrence, duration and severity of Long COVID in a sub-Saharan setting. **Global health impact for policy and action** Our findings underscore the potentially significant burden of long COVID in Kenya particularly among vulnerable populations. Results emphasize the need for integrating tailored support strategies for affected groups into routine healthcare and provide preliminary evidence for policymakers to establish dedicated long COVID care services for timely diagnosis and management.

## Background

The World Health Organization (WHO) estimates that 10–20% of the individuals may experience persistent symptoms after a ‘Severe Acute Respiratory Syndrome Coronavirus-2’ (SARS-CoV-2) infection, also known as post COVID-19 condition or long COVID [1]. Affected individuals include those who had initially mild symptoms or were asymptomatic at the time of initial SARS-CoV-2 infection [2,3]. Long COVID is defined by the WHO as the continuation or development of new symptoms three months after acute infection that last for at least two months and cannot be explained by an alternative cause [1,4]. Prevalence estimates of Long COVID widely vary due to differences in sample populations, study designs, and applied definitions of Long COVID [5]. Notably, data on long COVID from low- and middle-income countries (LMICs), including sub-Saharan African countries, are scarce and few reported cohort studies had a short follow-up period [6]. A longitudinal study conducted in South Africa reported that 39% of the participants had persistent COVID-19-related symptoms 6 months after infection, with fatigue being the most frequent symptom (32.1%) [7]. However, this study only followed patients for 6 months, hence the precise duration of post-COVID sequelae in these patients remained unclear. There is clearly an urgent unmet need for improved insights into the potential impact of long COVID in sub-Saharan African countries.

In the current study, we aimed to investigate the occurrence of persistent COVID-19-related symptoms, potential factors influencing the time to recovery, and risk factors for long COVID severity in a prospective cohort in the Nairobi Metropolitan region, the epicenter of the COVID-19 pandemic in Kenya. In addition, we specifically focused on factors associated with experiencing prolonged fatigue, representing the most commonly reported symptom of long COVID. Finally, we assessed the quality of life of participants at 12-month of follow-up.

## Methods

This study is reported according to the Strengthening the Reporting of Observational Studies in Epidemiology (STROBE) Statement for cohort studies [8].

### Study design and enrollment

This was a prospective cohort study of individuals with a positive polymerase chain reaction (PCR) result for SARS-CoV-2 at the Kenyatta University Teaching, Referral & Research Hospital (KUTRRH) and the Kenya Medical Research Institute (KEMRI). Eligible participants were enrolled between 8 February 2022 and 21 February 2023 and followed until 30 June 2023. Individuals could join the study at any time after a positive PCR test (i.e. open enrolment). Inclusion criteria included age ≥18 years old, sufficient understanding of English or Kiswahili, and living in the Nairobi metropolitan area. Exclusion criteria were inability to comply with the study procedures as evaluated by the recruiting study team and living in long-term facilities. Although the exclusion criteria included inability to comply with the study procedures and living in long-term care facilities, no participant met these criteria during the enrollment period.

The study team contacted individuals with a positive PCR test between December 2020 and July 2022 by telephone and distributed study flyers at KUTRRH. Participants provided written informed consent at enrollment. Participants were enrolled at a median of 99 days after testing positive for SARS-CoV-2. The study focus on long-term health outcomes ensured that persistent symptoms beyond acute phase of infection could adequately be assessed. Participants were asked specifically to recall symptoms since COVID-19 diagnosis or since the last follow-up visit. Potential recall bias introduced by delay in enrollment was assessed by conducting sensitivity analyses stratified by time since positive PCR and findings remained consistent across groups, suggesting minimal impact on validity of reported symptoms persistence.

### Study procedures and data collection

Enrollment and follow-up took place at the KUTRRH. Participants were reimbursed for travel costs. Trained research assistants conducted study procedures and collected data via SurveyCTO tablets. Data collected at enrollment included age (years), sex, marital status, education level, occupation, socioeconomic status, self-reported health conditions (asthma, hypertension, diabetes mellitus, dyslipidemia, Tuberculosis, HIV, cancer), anthropometric measurements (height, weight), blood pressure, self-reported COVID-19 vaccination status, acute COVID-19 treatment (baricitinib, tocilizumab, remdesivir, dexamethasone), hospital admission, and oxygen therapy. The primary outcome was the presence of COVID-19-related symptoms measured using the identity section of the Illness Perception Questionnaire (IPQ-R) [9]. The IPQ-R identity section consists of 14 symptoms (any pain, sore throat, nausea, breathlessness, weight change, fatigue, stiff joints, sore eyes, wheeziness, headaches, upset stomach, sleep difficulties, dizziness, loss of strength). Participants were asked whether they experienced symptoms since their SARS-CoV-2 infection and whether they believed these symptoms were related to COVID-19 (Supplementary Methods 1). Responses were categorized as having no symptoms and having ≥ 1 COVID-19-related symptoms. The long COVID severity was defined according to the total number of COVID-19-related symptoms reported. Due to the absence of standardized criteria for assessing long COVID severity, we assumed that a higher number of COVID-19-related symptoms corresponded to greater severity. The rationale is that a higher number of symptoms is often associated with greater functional impairment, worse health-related quality of life (HRQoL) and increased health care utilization. The secondary outcomes were fatigue [10] and quality of life [11]. Fatigue was measured using the Fatigue Assessment Scale (FAS) which is a 10-item scale evaluating symptoms of chronic fatigue and categorized as no fatigue (FAS score < 22) and fatigue (FAS score ≥ 22) [10] (Supplementary methods 2). Quality of life was measured using the SF-36 comprising 36 questions that cover eight domains (i.e. general health, physical functioning, physical health, emotional health, social functioning, pain, energy/fatigue, and emotional well-being) [11]. The primary and secondary outcomes were measured at enrollment and every 3 months until 12 months, except for quality of life data collected at enrollment and 12 months only. A sample size of 168 symptomatic and 168 asymptomatic participants was estimated to detect a 20% difference in risk between mild/moderate and severe/critical SARS-CoV-2 infection severity (Supplementary Text 1).

### Definitions of study variables and outcomes

COVID-19 illness onset was defined as the date of a positive PCR test obtained from the testing centres. Complete recovery was defined as the first moment upon which a participant reported no COVID-19-related symptoms in the past two weeks. Time to recovery was defined as the difference in days between the date of a positive PCR result (i.e. COVID-19 infection onset) and recovery from COVID-19-related symptoms. The severity of initial COVID-19 infection was categorized into mild/moderate and severe/critical, according to the World Health Organization criteria [12]. Mild/moderate COVID-19 infection was defined as participants reported not having had hospital admissions and oxygen therapy due to COVID-19 as well as home-based care and oxygen therapy. Severe/critical COVID-19 infection was defined as participants reported having had hospital admission and/or needed oxygen therapy due to COVID-19. BMI was categorized as follows: underweight or normal weight (BMI <25 Kg/M^2^); overweight (BMI 25–30 Kg/M^2^), and obese (BMI >30 Kg/M^2^). A FAS score less than 22 indicates ‘normal’ (i.e. healthy), between 22 and 34 indicates mild-moderate fatigue, and 35 or more indicates severe fatigue [13]. In this study, the FAS score was categorized as no fatigue (FAS score < 22) and fatigue (FAS score ≥ 22) as there was a low number of participants with severe fatigue (*n* = 2). SF-36 scores range from 0 (worst) to 100 (best). Overall quality of life (QoL) scores were used to transform quality of life as poor QoL (overall QoL scores < 50) and good QoL (overall QoL scores ≥ 50) [14]. Loss of follow-up was defined as withdrawal from the study or two consecutive unavailability despite three attempts to establish contact. Severity of Long COVID was defined as the total number of COVID-19-related symptoms; with a higher number of symptoms indicating more severe long COVID.

### Statistical methods

Data were summarized as numbers and percentages for categorical variables, and as mean (SD) or median (IQR) for continuous variables, for the overall sample and by SARS-CoV-2 infection severity. Alluvial plots depicted the transitions between COVID-19-related symptoms over time and the frequency of individuals with new or relapsing symptoms. Incidence proportions of COVID-19-related symptoms since a positive PCR test were reported, representing the number of participants who ever reported symptoms among those still in follow-up at each time point since a positive PCR test.

Kaplan–Meier survival analysis estimated the proportion of participants experiencing ongoing COVID-19-related symptoms (i.e. not fully recovered), excluding asymptomatic participants at enrollment. Given participants were not enrolled in the study immediately after their positive PCR test, they became ‘at-risk’ upon enrollment (left-censored), with time measured in days since a positive PCR test. Thus, the at-risk period began on the positive PCR test day until recovery of COVID-19-related symptoms, loss-to-follow-up, death, or the last study visit, whichever came first. The analysis was stratified by SARS-CoV-2 infection severity. Potential factors influencing the time to recovery from COVID-19-related symptoms were explored using an univariable and multivariable Cox proportional hazard model. The choice of potential factors was based on the literature, including characteristics at enrollment (i.e. age, sex, marital status, education, employment status, occupation, socioeconomic status, comorbidities, and vaccination) [15]. Variables with a p-value <0.25 in the univariable analysis were included in the multivariable model to avoid overfitting and improve generalizability [16]. Unadjusted hazard ratio (HR) and adjusted hazard ratio (aHR) and their corresponding 95%CIs are presented. We assessed each HR using the proportional-hazards assumptions test based on Schoenfeld residuals and checked the validity of the proportional hazards assumption using stcoxkm [17] (Supplementary Methods 3).

Poisson generalized estimating equation (GEE) models were developed to assess risk factors associated with long COVID severity [18]. GEE models included the total number of persistent COVID-19-related symptoms as an outcome and days since a positive PCR test, age, and sex as fixed covariates. Manual backward selection was used to retain variables in the model [16]. Variables included in the multivariable model were selected based on the p-value of < 0.25 in the univariable analysis and clinical plausibility. Variables were included in the multivariable model in this order: sociodemographic and clinical characteristics; SARS-CoV-2 infection severity; and vaccination status. Adjusted incidence risk ratio (aIRR) and their 95%CIs are presented. We investigated interactions between BMI and other variables (i.e. comorbidity, age) as obesity clusters with other comorbidities [19]. We addressed missing data using multiple imputation with chained equations (MICE) assuming missing at random (MAR) mechanism. The imputation model included all the variables that would be included in the substantive model. We created 50 multiply imputed datasets and estimates pooled using Rubin combination rules to ensure valid statistical inference. We reported both results from complete case analysis and MI with point estimates remaining largely consistent between the two approaches.

A binomial GEE logistic model was conducted as a secondary analysis to assess the presence of fatigue over time using a similar development strategy as the Poisson GEE model. Results are presented as odds ratio (OR) and their 95%CIs. Fatigue was used as a proxy for long COVID as fatigue is the most frequently reported persistent COVID-19-related symptom [20]. A linear regression was performed cross-sectionally to assess the association between long COVID and quality of life at 12 months of follow-up, as suggested by existing literature [21]. All analyses were performed using the Stata software (StataCorp, version 17.0).

## Results

### Study participants

A total of 2145 individuals were screened (Supplementary Figure S1), of whom 750 had a positive PCR test for SARS-CoV-2 infection. Of these, 459 individuals declined to participate (median age 43 (IQR = 31–61), 54% female) and 291 participants were enrolled in the study (Supplementary Table S1a, Figure S1). The median age of participants was 34 (IQR = 29–42) years and 172 (59.1%) were female (Table 1). The median time between a positive PCR test and enrollment into the study was 99 days (IQR = 71–169) with 113 of 291 (38.8%) participants enrolled less than 90 days after a positive PCR test. Five deaths occurred during follow-up (2 participants with mild/moderate and 3 participants with severe/critical SARS-CoV-2 infection) due to underlying comorbidity (HIV, diabetes, and hypertension complications).

**Table 1.** Characteristics of the participants of the long COVID study by SARS-CoV-2 infection severity at diagnosis.

Characteristic	Overall	Mild/Moderate	Severe/Critical
	N = 291	N = 249	N = 42
Age (median (IQR))	34 (29–42)	32 (29–39)	57 (40–63)
Age group (Years)			
<40	198 (68.0)	188 (75.5)	10 (23.8)
40–64	79 (27.1)	57 (22.9)	22 (52.4)
≥65	14 (4.8)	4 (1.6)	10 (23.8)
Sex			
Female	172 (59.1)	146 (58.6)	26 (61.9)
Male	119 (40.9)	103 (41.4)	16 (38.0)
Marital status			
Married	167 (57.4)	140 (56.2)	27 (64.3)
Never Married or Co-habited	97 (33.3)	92 (37.0)	5 (11.9)
Divorced with a living partner	18 (6.2)	14 (5.6)	4 (9.5)
Partner deceased	9 (3.1)	3 (1.2)	6 (14.3)
Education level			
None or Primary	27 (9.3)	18 (7.2)	9 (21.4)
Secondary	48 (16.5)	36 (14.5)	12 (28.6)
Tertiary	216 (74.2)	195 (78.3)	21 (50.0)
Employed	250 (85.9)	225 (90.4)	25 (59.5)
Self-reported socioeconomic status^#^			
Low class	32 (11.0)	25 (10.0)	7 (16.7)
Middle class	158 (54.3)	142 (58.0)	16 (38.0)
Upper class	68 (23.4)	55 (22.1)	13 (31.0)
Unknown	33 (11.3)	27 (10.9)	6 (14.3)
Healthcare professionals	112 (38.5)	110 (44.2)	2 (4.8)
BMI, Kg/M^2^			
Normal weight (BMI <25 Kg/M^2^)	126 (43.3)	109 (43.8)	17 (40.5)
Overweight (BMI 25–30 Kg/M^2^)	95 (32.6)	89 (35.7)	6 (14.3)
Obesity (BMI >30 Kg/M^2^)	70 (24.1)	51 (20.5)	19 (45.2)
Comorbidities			
None	219 (75.3)	204 (81.9)	15 (35.7)
One or more	72 (24.7)	45 (18.1)	27 (64.3)
Asthma	14 (4.8)	11 (4.4)	3 (7.1)
Hypertension	37 (12.7)	20 (8.0)	17 (40.5)
Diabetes Mellitus	17 (5.8)	4 (1.6)	13 (31.0)
Dyslipidaemia	13 (4.5)	8 (3.2)	5 (11.9)
Tuberculosis	9 (3.1)	8 (3.2)	1 (2.4)
HIV	4 (1.4)	3 (1.2)	1 (2.4)
Cancer	2 (0.7)	1 (0.4)	1 (2.4)
Symptom status at first study visit			
Symptomatic	187 (64.3)	152 (61.0)	35 (83.3)
Asymptomatic	104 (35.7)	97 (39.0)	7 (16.7)
Fatigue at first study visi^t*^	60 (20.6)	43 (17.3)	17 (40.5)
Good Quality of life (QoL) at first study visit^$^	246 (84.5)	220 (88.3)	26 (61.9)
COVID-19 vaccination prior to enrollment	255 (87.6)	226 (90.8)	29 (69.1)
Covid-19 vaccine type			
AstraZeneca	197 (77.3)	176 (77.8)	21 (72.4)
Moderna	28 (11.0)	26 (11.5)	2 (6.9)
Janssen J & J	18 (7.0)	13 (5.8)	5 (17.2)
Pfizer	12 (4.7)	11 (4.9)	1 (3.5)
Received medicine to treat COVID-19	33 (11.3)	12 (4.8)	21 (50.0)
Types of medicine to treat COVID-19			
Tocilizuma	12 (4.1)	1 (14.3)	11 (52.4)
Remdesivir	1 (0.3)	0 (0.0)	1 (2.4)
Dexamethasone	15 (5.2)	6 (2.4)	9 (21.4)
Other long-term medication	8 (2.7)	6 (2.4)	2 (4.8)
Admitted to the hospital and put on oxygen	42 (14.4)	0 (0.0)	42 (100.0)
Home-based care and receiving oxygen	6 (2.1)	2 (0.8)	4 (9.5)
Time from + PCR to first study visit (Median (IQR))	99 (71–169)	99 (71–167)	104 (75–171)

IQR: Interquartile Range. Obesity: body mass index >30 Kg/M^2^. HIV: Human immunodeficiency virus. PCR: Polymerase chain reaction. QoL: Quality of life. SES: Socioeconomic status.

Continuous variables are presented as median (IQR); categorical and binary variables are presented as N (%) and compared using the Pearson X^2^ test.

Clinical severity groups are defined as follows: Mild/moderate COVID-19 infection was defined as participants reported not having had hospital admissions and oxygen therapy due to COVID-19 as well as home-based care and oxygen therapy and severe otherwise. Severe/critical COVID-19 infection was defined as participants reported having had hospital admission and/or needed oxygen therapy due to COVID.

*Fatigue: defined as no fatigue (FAS score < 22) and Fatigue (FAS score score ≥22)

^$^Quality of life (QoL): defined as poor QoL (overall score < 50) and good QoL (overall score ≥ 50).

SES was measured using a ladder representing socioeconomic standing of people in the community. At the top were those who are best off, have most money, most education and most respected jobs. At the bottom are people who have least money and least jobs.

^#^The total did not equal 291 due to missing data.

### Incidence proportions of COVID-19-related symptoms

The most frequently reported COVID-19-related symptoms at enrollment were fatigue, pain, sore throat, headaches, and loss of strength at 40.2%, 36.8%, 36.8%, 36.4%, and 31.6%, respectively (Supplementary Table S1b; Supplementary Figure S2). At 3 months since a positive PCR test, fatigue (48.7%), pain (44.2%), headaches (44.2%), sore throat (40.7%), and loss of strength (40.7%) were still commonly reported symptoms. Fatigue, stiff joints, wheeziness, headaches, sleep difficulties, dizziness, and loss of strength were more frequently reported in the severe/critical infection group compared to the mild/moderate infection group (Table 2). The proportion of participants with at least one COVID-19-related symptom at 6, 12, 18, and 24 months since a positive PCR test was 53.1%, 33.5%, 39.7%, and 29.4%, respectively. Alluvial plots indicated that while most of the participants transitioned to a lower number of symptoms over time, some developed new symptoms (Supplementary Figure S3).

**Table 2.** Incidence proportion of 14 COVID-19-related symptoms since a positive PCR by SARS-CoV-2 infection severity.

Since positive PCR*	Month 3				Month 6					Month 9				Month 12			
Severity group	Overall	Mild/Moderate	Severe/Critical	P-Value	Overall		Mild/Moderate	Severe/Critical	P-Value	Overall	Mild/Moderate	Severe/Critical	P-Value	Overall	Mild/Moderate	Severe/Critical	*P*-Value
N→	N = 113	N = 100	N = 13		N = 162		N = 137	N = 25		N = 197	N = 167	N = 30		N = 215	N = 186	N = 29	
Symptom ↓																	
Pain	50 (44.2)	46 (46.0)	4 (30.8)	0.298	51 (31.5)		42 (30.7)	9 (36.0)	0.597	22 (11.2)	15 (9.0)	7 (23.3)	0.022	28 (13.0)	19 (10.2)	9 (31.0)	0.002
Sore throat	46 (40.7)	42 (42.0)	4 (30.8)	0.438	52 (32.1)		46 (33.6)	6 (24.0)	0.346	26 (13.2)	21 (12.6)	5 (16.7)	0.542	29 (13.5)	24 (12.9)	5 (17.2)	0.525
Nausea	23 (20.4)	18 (18.0)	5 (38.5)	0.085	22 (13.6)		18 (13.1)	4 (16.0)	0.701	15 (7.6)	11 (6.6)	4 (13.3)	0.2	11 (5.1)	8 (4.3)	3 (10.3)	0.169
Breathlessness	30 (26.5)	23 (23.0)	7 (53.9)	0.018	36 (22.2)		29 (21.2)	7 (28.0)	0.45	21 (10.7)	14 (8.4)	7 (23.3)	0.015	17 (7.9)	9 (4.8)	8 (27.6)	<0.001
Weight loss	13 (11.5)	9 (9.0)	4 (30.8)	0.021	21 (13.0)		16 (11.7)	5 (20.0)	0.255	14 (7.1)	9 (5.39)	5 (16.7)	0.027	6 2.8)	4 (2.2)	2 (6.9)	0.149
Fatigue	55 (48.7)	47 (47.0)	8 (61.5)	0.324	51 (31.5)		41 (29.9)	10 (40.0)	0.319	36 (18.3)	25 (15.0)	11 (36.7)	0.005	27 (12.6)	18 (9.7)	9 (31.0)	0.001
Stiff joints	28 (24.8)	22 (22.0)	6 (46.2)	0.058	36 (22.2)		28 (20.4)	8 (32.0)	0.201	23 (11.7)	13 (7.8)	10 (33.3)	<0.001	20 (9.3)	14 (7.5)	6 (20.7)	0.023
Sore eyes	14 (12.4)	13 (13.0)	1 (7.7)	0.585	21 (13.0)		18 (13.1)	3 (12.0)	0.876	13 (6.6)	12 (7.2)	1 (3.3)	0.434	12 (5.6)	8 (4.3)	4 (13.8)	0.038
Wheeziness	18 (15.9)	15 (15.0)	3 (23.1)	0.454	23 (14.2)		18 (13.1)	5 (20.0)	0.366	17 (8.6)	12 (7.2)	5 (16.7)	0.089	7 (3.3)	3 (1.6)	4 (13.8)	0.001
Headaches	50 (44.2)	42 (42.0)	8 (61.5)	0.182	52 (32.1)		46 (33.6)	6 (24.0)	0.346	25 (12.7)	20 (12.0)	5 (16.7)	0.477	28 (13.0)	23 (12.4)	5 (17.2)	0.468
Upset stomach	16 (14.2)	15 (15.0)	1 (7.7)	0.477	16 (9.9)		11 (8.0)	5 (20.0)	0.065	6 (3.0)	5 (3.0)	1 (3.3)	0.921	8 (3.7)	5 (2.7)	3 (10.3)	0.043
Sleep difficulties	25 (22.1)	22 (22.0)	3 (23.1)	0.93	33 (20.4)		26 (19.0)	7 (28.0)	0.303	15 (7.6)	8 (4.8)	7 (23.3)	<0.001	15 (7.0)	9 (4.8)	6 (20.7)	0.002
Dizziness	30 (26.5)	24 (24.0)	6 (46.2)	0.089	26 (16.0)		23 (16.8)	3 (12.0)	0.549	18 (9.1)	11 (6.6)	7 (23.3)	0.003	15 (7.0)	9 (4.8)	6 (20.7)	0.002
Loss of strength	46 (40.7)	39 (39.0)	7 (53.9)	0.305	36 (22.2)		27 (19.7)	9 (36.0)	0.072	25 (12.7)	16 (9.6)	9 (30.0)	0.002	13 (6.0)	9 (4.8)	4 (13.8)	0.06
Overall symptoms	82 (72.6)	70 (70.0)	12 (92.3)	0.09	86 (53.1)		69 (50.4)	17 (68.0)	0.104	70 (35.5)	55 (32.9)	15 (50.0)	0.072	72 (33.5)	55 (29.6)	17 (58.6)	0.002
Since positive PCR	Month 15				Month 18					Month 21				Month ≥24			
Severity group	Overall	Mild/Moderate	Severe/Critical	P-Value		Overall	Mild/Moderate	Severe/Critical	P-Value	Overall	Mild/Moderate	Severe/Critical	P-Value	Overall	Mild/Moderate	Severe/Critical	P-Value
N→	N = 206	N = 179	N = 27			N = 194	N = 168	N = 26		N = 83	N = 73	N=10		N = 34	N = 30	N = 4	
Symptom ↓																	
Pain	25 (12.1)	19 (10.6)	6 (22.2)	0.085		35 (18.0)	26 (15.5)	9 (34.6)	0.018	10 (12.0)	9 (12.3)	1 (10.0)	0.832	5 (14.7)	4 (13.3)	1 (25.0)	0.536
Sore throat	29 (14.1)	23 (12.9)	6 (22.2)	0.192		36 (18.6)	31 (18.5)	5 (19.2)	0.924	5 (6.0)	4 (5.5)	1 (10.0)	0.573	3 (8.8)	3 (10.0)	0 (0.0)	0.508
Nausea	6 (2.9)	5 (2.8)	1 (3.7)	0.793		15 (7.7)	11 (6.6)	4 (15.4)	0.116	3 (3.6)	3 (4.1)	0 (0.0)	0.514	1 (2.9)	1 (3.3)	0 (0.0)	0.711
Breathlessness	21 (10.2)	14 (7.8)	7 (25.9)	0.004		27 (13.9)	21 (12.5)	6 (23.1)	0.147	9 (10.8)	8 (11.0)	1 (10.0)	0.927	1 (2.9)	1 (3.3)	0 (0.0)	0.711
Weight loss	7 (3.4)	6 (3.4)	1 (3.7)	0.925		16 (8.2)	14 (8.3)	2 (7.7)	0.912	1 (1.2)	1 (1.4)	0 (0.0)	0.71	0 (0.0)	0 (0.0)	0 (0.0)	-
Fatigue	26 (12.6)	21 (11.7)	5 (18.5)	0.322		41 (21.1)	34 (20.2)	7 (26.9)	0.437	6 (7.2)	6 (8.2)	0 (0.0)	0.347	5 (14.7)	4 (13.3)	1 (25.0)	0.536
Stiff joints	11 (5.3)	8 (4.5)	3 (11.1)	0.152		21 (10.8)	17 (10.1)	4 (15.4)	0.421	3 (3.6)	3 (4.1)	0 (0.0)	0.514	1 (2.9)	0 (0.0)	1 (25.0)	0.005
Sore eyes	4 (1.9)	2 (1.1)	2 (7.4)	0.027		14 (7.2)	10 (6.0)	4 (15.4)	0.084	2 (2.4)	2 (2.7)	0 (0.0)	0.596	1 (2.9)	1 (3.3)	0 (0.0)	0.711
Wheeziness	6 (2.9)	2 (1.1)	4 (14.8)	<0.001		6 (3.1)	5 (3.0)	1 (3.9)	0.812	2 (2.4)	1 (1.4)	1 (10.0)	0.095	3 (8.8)	1 (3.3)	2 (50.0)	0.002
Headaches	25 (12.1)	22 (12.3)	3 (11.1)	0.861		35 (18.0)	31 (18.5)	4 (15.4)	0.705	5 (6.0)	5 (6.9)	0 (0.0)	0.393	3 (8.8)	3 (10.0)	0 (0.0)	0.508
Upset stomach	6 (2.9)	4 (2.2)	2 (7.4)	0.136		8 (4.1)	6 (3.6)	2 (7.7)	0.325	3 (3.6)	2 (2.7)	1 (10.0)	0.249	3 (8.8)	3 (0.0)	0 (0.0)	0.508
Sleep difficulties	18 (8.7)	12 (6.7)	6 (22.2)	0.008		18 (9.3)	11 (6.6)	7 (26.9)	0.001	3 (3.6)	3 (4.1)	0 (0.0)	0.514	2 (5.9)	2 (6.7)	0 (0.0)	0.595
Dizziness	18 (8.7)	12 (6.7)	6 (22.2)	0.008		18 (9.3)	13 (7.7)	5 (19.2)	0.06	2 (2.4)	2 (2.7)	0 (0.0)	0.596	3 (8.8)	3 (10.0)	0 (0.0)	0.508
Loss of strength	18 (8.7)	12 (6.7)	6 (22.2)	0.008		24 (12.4)	18 (10.7)	6 (23.1)	0.075	3 (3.6)	3 (4.1)	0 (0.0)	0.514	3 (8.8)	2 (6.7)	1 (25.0)	0.225
Overall symptoms	65 (31.6)	47 (26.3)	18 (66.7)	<0.001		77 (39.7)	61 (36.3)	16 (61.5)	0.014	18 (21.7)	15 (20.6)	3 (30,0)	0.496	10 (29.4)	8 (26.7)	2 (50.0)	0.336

*The median time between a positive PCR and enrollment into the study was 99 days (IQR = 71–169) and 38.8% (*n* = 113) out of 291 participants had less than 90 days between a positive PCR test and first visit.

### Time to recovery from COVID-19-related symptoms

**Figure 1.**
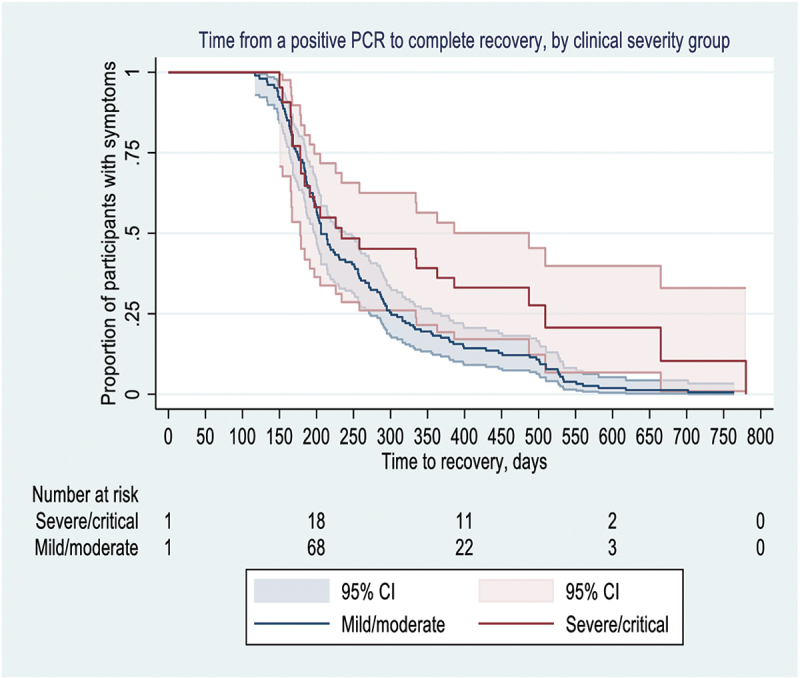
Kaplan Meier curve representing the proportion of participants with COVID-19- related symptoms (y-axis) in days between a positive PCR and complete recovery (x-axis), stratified by SARS-CoV-2 infection (serve/critical vs mild/moderate).

A total of 171 participants were symptomatic at enrollment. Time to recovery was significantly longer in individuals with severe/critical acute COVID-19 than in those with mild/moderate COVID-19 (*p* = 0.016, Log-rank test) (Figure 1). Among those with mild/moderate SARS-CoV-2 infection, the median time to complete recovery was 206 days (95%CI, 198; 239), while for those with severe/critical illness, the median time to recovery was 234 days (95%CI, 178; 487).

A total of 171 symptomatic participants were included in the survival analysis. As participants were not followed immediately after their positive PCR test, follow-up was left-censored, with participants recovering from COVID-19-related symptoms during the 2 years after a positive PCR. Shaded areas represent a 95% confidence interval (CI). The number of study participants at risk since positive PCR is below the graph. COVID-19; coronavirus disease 2019; SARS-CoV-2: Severe Acute Respiratory Syndrome Coronavirus 2.

### Factors influencing the time to recovery from COVID-19-related symptoms

There was no significant difference between sexes in time to recovery (aHR = 0.787 [95%CI, 0.573; 1.082). Participants aged 40–64 years experienced 36.5% slower recovery than those aged <40 years (aHR = 0.635 [95%CI, 0.429; 0.941]). Participants with tertiary education (aHR = 1.869 [95%CI, 1.050; 3.327]) recovered almost twice as rapidly as those with primary education (Table 3).

**Table 3.** Cox proportional hazard model of factors influencing the time to recovery from COVID-19-related symptoms (n = 171).

Univariable Cox proportional hazard model			Multivariable Cox proportional hazard model		
Factors	HR (95% CI)	*p*-value	Factors	aHR (95% CI)	*p*-value
Sex		0.430	Sex		0.160
Male	Ref		Male	Ref	
Female	0.888 (0.662 1.192)		Female	0.787 (0.573 1.082)	
Age group (Years)		0.003	Age group (Years)		0.054
<40	Ref		<40	Ref	
40–64	0.566 (0.385 0.832)		40–64	0.635 (0.429 0.941)	
≥65	0.474 (0.185 1.213)		≥65	0.687 (0.214 2.202)	
Education level		0.002	Education level		0.011
Primary	Ref		Primary	Ref	
Secondary	1.244 (0.652 2.373)		Secondary	1.258 (0.66 2.396)	
Tertiary	2.042 (1.16 3.593)		Tertiary	1.869 (1.05 3.327)	
BMI, Kg/m2		<0.001	BMI, Kg/m2		0.256
Normal	Ref		Normal	Ref	
Overweight	1.252 (0.892 1.757)		Overweight	1.097 (0.762 1.578)	
Obese	1.027 (0.697 1.512)		Obese	1.211 (0.819 1.791)	
Comorbidity		0.057	Comorbidity		0.618
None	Ref		None	Ref	
One or more	0.689 (0.469 1.012)		One or more	0.877 (0.551 1.395)	
Vaccination status		0.046	Vaccination status		0.230
Yes	Ref		Yes	Ref	
No	0.601 (0.365 0.99)		No	0.734 (0.44 1.226)	

HR = Hazard ratio; aHR = Adjusted hazard ratio estimated using a multivariable Cox proportional hazard model including sex, age group, education, body mass index, comorbidities and vaccination status.

### Risk factors associated with long COVID severity

Female participants were 1.4 times more likely than males to report a greater number of symptoms (aIRR = 1.418 [95%CI, 1.078; 1.864]). Individuals with tertiary education were less likely to report a higher number of COVID-19-related symptoms compared to those with primary education (aIRR = 0.489 [95%CI, 0.415; 0.576]). Having one or more comorbidities leads to a higher risk of reporting a greater number of COVID-19-related symptoms over time compared to having no comorbidity (aIRR = 2.415 [95%CI, 1.639; 3.559]). Individuals with mild/moderate initial COVID-19 severity are less likely to report a higher number of COVID-19-related symptoms compared to those with severe/critical severity (aIRR = 0.806 (95%CI, 0.701; 0.926). On average, with each additional day since a positive PCR test, participants experienced a small but statistically significant reduction in the risk of reporting a greater number of COVID-19-related symptoms (aIRR = 0.999 [95%CI, 0.997; 1.000]) (Table 4). Multiple imputation results showed consistent results except for the association between COVID-19 clinical and symptom burden.

**Table 4.** Poisson GEE multivariable model on risk factors associated with long COVID severity over time.

Any COVID-19 related symptoms (counts)				
	Complete Case (CC)		Multiple Imputation (MI)	
	N = 228		N = 291	
Factors (Selected prior)	aIRR (95% CI)	*P*-value	aIRR (95% CI)	*P*-value
Sex		0.007		0.012
Male	Ref			
Female	1.404 (1.119 1.762)		1.418 (1.078 1.864)	
Age group		0.372		0.194
<40	Ref			
40–64	0.957 (0.634 1.446)		0.932 (0.675 1.288)	
≥65	0.507 (0.304 0.846)		0.504 (0.333 0.764)	
Education		0.024		0.066
None/Primary	Ref			
Secondary	0.984 (0.747 1.296)		0.899 (0.748 1.081)	
Tertiary	0.554 (0.35 0.877)		0.489 (0.415 0.576)	
Employment		0.790		0.488
No	Ref			
Yes	0.841 (0.525 1.346)		1.41 (0.708 2.807)	
Socioeconomic status		0.003		0.671
Low class	Ref			
Middle	1.451 (0.788 2.671)		1.361 (0.709 2.613)	
Upper class	1.011 (0.196 5.215)		1.013 (0.211 4.872)	
BMI		0.053		0.831
Normal Weight	Ref			
Overweight	0.801 (0.359 1.783)		0.811 (0.412 1.597)	
Obese	0.807 (0.195 3.333)		0.852 (0.247 2.94)	
Comorbidity		<0.001		<0.001
None	Ref			
One or more	2.392 (1.503 3.807)		2.415 (1.639 3.559)	
Covid-19 Clinical severity^b^		0.422		0.014
Severe/critical	Ref			
Mild/Moderate	0.861 (0.737 1.006)		0.806 (0.701 0.926)	
Vaccination status		0.122		0.980
Vaccinated	Ref			
Unvaccinated	0.861 (0.737 1.006)		0.806 (0.701 0.926)	
Days	0.999 (0.998 1.000)	0.076	0.999 (0.997 1.000)	0.103

^a^IRR-Adjusted Incidence Risk Ratio: CI: Confidence interval.

^ab^SARS-CoV-2 infection severity groups are defined as follows: Mild/moderate SARS-CoV-2 infection was defined as participants who reported not having had hospital admissions and oxygen therapy due to COVID-19 as well as home-based care and oxygen therapy and severe otherwise. Severe/critical SARS-CoV-2 infection was defined as participants reported having had hospital admission and/or needed oxygen therapy due to COVID-19.

^bc^Time since a positive PCR.

^c^Statistical significance is inferred at p < 0.05 and/or when the 95% confidence interval does not include the null value (1).

Complete Case (CC) observations = 1140 and Multiple Imputation observations = 1455

### Fatigue and quality of life

Fatigue was 1.78 more likely to be reported by females than males (aOR = 1.781 [95%CI, 1.128; 2.811]). The adjusted odds of reporting fatigue in employed individuals were about half (or 48% lower) than in unemployed individuals (aOR = 0.518 [95%CI, 0.273; 0.981]). Individuals with high socioeconomic status were 50% less likely to have fatigue compared with those with low socioeconomic status (aOR = 0.507 [95%CI, 0.316; 0.813]). Individuals with mild/moderate SARS-CoV-2 infection were 61% less likely to have fatigue compared to those with severe/critical (aOR = 0.390 [95%CI, 0.199; 0.764]). On average, with an additional day since a positive PCR test, participants experienced a small but statistically significant decrease in risk for fatigue (aOR = 0.999 [95%CI, 0.998; 1.000]) (Supplementary Table S2a).

Quality of life was significantly lower in individuals who experienced COVID-19-related symptoms at 12 months compared with those with no symptoms (aβ = -1.705 [95% CI, −2.595; −0.816]). Time since infection was not significantly associated with quality of life (*p* = 0.326) when adjusting for other factors (Supplementary Table S2b).

## Discussion

At 6- and 12-months post-SARS-CoV-2-infection, half and one-third of participants, respectively, still reported COVID-19-related symptoms, with fatigue being the most commonly reported complaint. These findings align with existing literature on long COVID [22,23]. Older individuals (40–64 years) had a slower recovery than those under 40, while participants with tertiary education recovered more quickly than those with primary education. Female participants, individuals with primary education, and those with one or more comorbidities were more likely to experience severe long COVID.

Time to recover from COVID-19-related symptoms was associated with older age, confirming other reports in the literature [24]. This may be due to age-related immune changes that increase susceptibility to infections and disease progression [25]. Due to the small number of participants aged 65 and older (*n* = 14), we lacked the statistical power to determine if slower recovery applied to this age group. Highly educated participants were more likely to recover faster from COVID-19-related symptoms, aligning with a Ghanaian study that found individuals with tertiary education had better recovery rates than those with lower education [26].

When examining risk factors for long COVID severity, we found that participants with tertiary education had a significantly lower risk of experiencing a higher number of symptoms compared with those with only primary education. This is consistent with findings from the literature as studies suggest that higher education predicts better health literacy, enabling easier access to healthcare [27]. High education often correlates with high socioeconomic status, enhancing the ability to take sick leave for recovery [28]. Indeed, in Kenya, higher education is typically associated with better socioeconomic status and comprehensive health insurance, easing medical costs, and improving access to healthcare [29,30]. This contrasts with Kenyan citizens who rely on the National Health Insurance Fund (NHIF) which, despite being the main health insurer, has limitations in coverage and quality of services [29,31]. Our findings highlight that higher education and socioeconomic status may directly and indirectly contribute to better outcomes such as recovery from COVID-19-related symptoms. The government’s reluctance to cover COVID-19 treatment costs and support vulnerable citizens, particularly low-income earners, may be linked to the relatively low number of reported cases in Kenya [29]. This suggests the possibility of a high number of unrecorded asymptomatic cases due to limited testing. To better respond to future disease outbreaks, the government should invest in expanding and strengthening health infrastructure, ensuring equitable access to testing and treatment across all economic and social groups.

Consistent with reports from other settings, female sex was also significantly associated with long COVID severity in our study [15]. Hypothesized mechanisms to explain the association between female sex and long COVID include hormones perpetuating the hyperinflammatory status of the acute phase of COVID-19 even after recovery [32] and immune dysfunction [33]. Also, consistent with the literature we found that having one or more comorbidities is associated with increased risk for long COVID severity [7,34]. This could be due to immunosuppression caused by underlying health conditions, making them susceptible to severe infections and prolonged recovery periods [35]. In addition, many comorbidities are linked to chronic inflammation resulting in more severe inflammation [36]. However, we also recognize that participants may have reported symptoms related to pre-existing comorbidities during follow-up, and therefore we are uncertain to what extent the association between having comorbidities and severity of long COVID is influenced by reporting bias.

In addition to exploring the duration and severity of Long COVID, we also specifically focused on fatigue, representing the most commonly reported Long COVID symptom. We found that post-COVID fatigue affects females more severely than males [37] and participants with severe SARS-CoV-2 infection reported more pronounced fatigue [38] than individuals with milder infections. Additionally, participants with higher socioeconomic status and those who are employed reported less fatigue compared to those from low socioeconomic backgrounds [38,39]. This is likely due to better access to healthcare, resources for recovery, and less exposure to stressors that can exacerbate fatigue, as noted previously. Finally, individuals with COVID-19-related symptoms were more likely to report poor QoL. This may be driven by the activity impairment, disability, and work-life disruption resulting from Long COVID, as previously implicated by other studies [40], and suggests a substantial psychosocial burden associated with Long COVID that may have profound socio-economic consequences if not addressed.

The comparison of results with other sub-Saharan countries was hampered due to limited research on long COVID in African populations [6]. Interestingly, the overall risk factors identified in this study were similar to those found in high-income countries (HICs). Since the current study’s young, highly educated population (‘healthy volunteer effect’) may indeed be comparable to populations studied in HICs. Further research is needed to understand long COVID in more rural areas of Kenya and among lower socioeconomic groups with limited access to care.

Based on our knowledge, this study is the first in Kenya to report long-term health outcomes following a SARS-CoV-2 infection. However, the study had several limitations. The convenience sample introduced selection bias, hampering the generalization of results to the population with long COVID in Kenya. SARS-CoV-2 PCR test costs were not covered by NHIF leading to a reduced sample size skewed towards highly educated and middle-class individuals. We suggest that future studies should use alternative recruitment strategies to capture a more diverse population. Participants could join at any time since acute SARS-CoV-2 infection, with many enrolling ≥6 months after a positive PCR test, which introduces potential confounding. To address this, analyses were adjusted for time since the positive PCR test. Since participants were enrolled 99 days after testing positive for SARS-CoV-2, individuals with ongoing symptoms may have likely participated in the study introducing selection bias. This might have led to overestimated prevalence of long-term symptoms. The reliance on self-reported symptoms to define long COVID may have also led to bias. We tried to minimize that by adjusting the models for other comorbidities. While our study identified significant associations of certain risk factors and long COVID, these findings should be interpreted with caution regarding causality. Given the nature of our study, residual confounding and unmeasured variables may influence the observed associations. The absence of a control group may limit our ability to account for potential confounders. To partially address this limitation, we asked participants whether their symptoms were related to COVID-19 and adjusted the models for reports comorbidities. We only included symptoms that participants attributed to COVID-19. However, future studies should ideally include a control group of infected individuals without persistent COVID-19-related symptoms to understand the effect of long COVID. Loss-to-follow-up affected 25% of the sample, despite mitigation strategies (i.e. phone reminders and transportation support). We partially handled this by multiply imputing data that was missing and the results were consistent with complete case analysis, except that we had more power to find the association between COVID-19 clinical severity and symptom burden.

## Conclusion

Six months after infection, half of the participants still had COVID-19-related symptoms, and a third continued to experience them after a year, with fatigue being the most common symptom. Faster recovery time from symptoms was associated with younger age and higher education level. Severe long COVID was more likely in women and individuals with lower levels of education or pre-existing health conditions. Our findings underscore the potentially significant burden of long COVID in Kenya, and the need to develop tailored support strategies for specific affected groups, particularly among vulnerable communities.

## Supplementary Material

Supplementary files.docx

## Preprint

MedRxiv server for health sciences, doi: https://doi.org/10.1101/2024.11.21.24316116

## References

[1] WHO. Post COVID-19 condition (long COVID). 2022 [cited 2023 Nov 24]. Available from: https://www.who.int/europe/news-room/fact-sheets/item/post-covid-19-condition

[2] Michelen M, Manoharan L, Elkheir N, et al. Characterising long COVID: a living systematic review. BMJ Glob Health. 2021;6:e005427. doi: 10.1136/bmjgh-2021-005427 PMC8478580 34580069

[3] Huang Y, Pinto MD, Borelli JL, et al. COVID symptoms, symptom clusters, and predictors for becoming a Long-Hauler looking for clarity in the haze of the pandemic. Clin Nurs Res. 2022;31:1390–13. doi: 10.1177/10547738221125632 36154716 PMC9510954

[4] Soriano JB, Murthy S, Marshall JC, et al. A clinical case definition of post-COVID-19 condition by a Delphi consensus. Lancet Infect Dis. 2022;22:e102–7. doi: 10.1016/S1473-3099(21)00703-9 34951953 PMC8691845

[5] Davis HE, McCorkell L, Vogel JM, et al. Long COVID: major findings, mechanisms and recommendations. Nat Rev Microbiol. 2023;21:133–146. doi: 10.1038/s41579-022-00846-2 36639608 PMC9839201

[6] Frallonardo L, Segala FV, Chhaganlal KD, et al. Incidence and burden of long COVID in Africa: a systematic review and meta-analysis. Sci Rep. 2023;13:21482. doi: 10.1038/s41598-023-48258-3 38057338 PMC10700349

[7] Jassat W, Mudara C, Vika C, et al. A cohort study of post-COVID-19 condition across the beta, delta, and omicron waves in South Africa: 6-month follow-up of hospitalized and nonhospitalized participants. Int J Infect Dis. 2023;128:102–111. doi: 10.1016/j.ijid.2022.12.036 36587841 PMC9800016

[8] Von Elm E, Altman DG, Egger M, et al. G0tzsche PC, Vandenbroucke JP; STROBE initiative. The strengthening the reporting of observational studies in epidemiology (STROBE) statement: guidelines for reporting observational studies. Ann Intern Med. 2007;147:573–577. doi: 10.7326/0003-4819-147-8-200710160-00010 17938396

[9] Moss-Morris R, Weinman J, Petrie K, et al. The revised illness perception questionnaire (IPQ-R). Psychol Health. 2002;17:1–16. doi: 10.1080/08870440290001494

[10] Shahid A, Wilkinson K, Marcu S, et al. Fatigue assessment scale (FAS). STOP, THAT and one hundred other sleep scales. 2012:161–162. doi: 10.1007/978.

[11] Brazier E, Harper R, Jones NMB, et al. Validating the SF-36 health survey questionnaire: new outcome measure for primary care. BMJ. 1992;305:160–164. doi: 10.1136/bmj.305.6846.160 1285753 PMC1883187

[12] WHO. COVID-19 symptoms and severity [internet]. 2023 [cited 2023 Nov 24]. Available from: https://www.who.int/westernpacific/emergencies/covid-19/information/asymptomatic-covid-19

[13] Hendriks C, Drent M, Elfferich M, et al. The fatigue assessment scale: quality and availability in sarcoidosis and other diseases. Curr Opin Pulm Med. 2018;24:495–503. doi: 10.1097/MCP.0000000000000496 29889115

[14] Framework IC, Sherbourne CD. The MOS 36-item short-form health survey (SF-36). Med Care. 1992;30:473–483. doi: 10.1097/00005650-199206000-00002 1593914

[15] Tsampasian V, Elghazaly H, Chattopadhyay R, et al. Risk factors associated with Post−COVID-19 condition. JAMA Intern Med. 2023;183:566. doi: 10.1001/jamainternmed.2023.0750 36951832 PMC10037203

[16] Harrell FE Jr, Lee KL, Mark DB. Multivariable prognostic models: issues in developing models, evaluating assumptions and adequacy, and measuring and reducing errors. Stat Med. 1996;15:361–387. doi: 10.1002/(SICI)1097-0258(19960229)15:4<361::AID-SIM168>3.0.CO;2-4 8668867

[17] Ruhe C. Estimating survival functions after stcox with time-varying coefficients. Stata J. 2016;16:867–879. doi: 10.1177/1536867X1601600404

[18] Twisk JWR. Applied longitudinal data analysis for epidemiology: a practical guide. UK: Cambridge University Press; 2013.

[19] Kivimäki M, Strandberg T, Pentti J, et al. Body-mass index and risk of obesity-related complex multimorbidity: an observational multicohort study. Lancet Diabetes Endocrinol. 2022;10:253–263. doi: 10.1016/S2213-8587(22)00033-X 35248171 PMC8938400

[20] Marjenberg Z, Leng S, Tascini C, et al. Risk of long COVID main symptoms after SARS-CoV-2 infection: a systematic review and meta-analysis. Sci Rep. 2023;13:15332. doi: 10.1038/s41598-023-42321-9 37714919 PMC10504382

[21] Carlile O, Briggs A, Henderson AD, et al. Impact of long COVID on health-related quality-of-life: an OpenSAFELY population cohort study using patient-reported outcome measures (OpenPROMPT). Lancet Reg Health–Eur. 2024;40:100908. 38689605 10.1016/j.lanepe.2024.100908 PMC11059448

[22] Nyasulu PS, Tamuzi JL, Erasmus RT. Burden, causation, and particularities of long-COVID in African populations: a rapid systematic review. IJID Regions. 2023;8:137–144. doi: 10.1016/j.ijregi.2023.08.004 37674565 PMC10477483

[23] Nalbandian A, Sehgal K, Gupta A, et al. Post-acute COVID-19 syndrome. Nat Med. 2021;27:601–615. doi: 10.1038/s41591-021-01283-z 33753937 PMC8893149

[24] Tolossa T, Wakuma B, Seyoum Gebre D, et al. Time to recovery from COVID-19 and its predictors among patients admitted to treatment center of Wollega University Referral Hospital (WURH), Western Ethiopia: survival analysis of retrospective cohort study. PLOS ONE. 2021;16:e0252389. doi: 10.1371/journal.pone.0252389 34111146 PMC8191892

[25] Bajaj V, Gadi N, Spihlman AP, et al. Aging, immunity, and COVID-19: how age influences the host immune response to coronavirus infections? Front Physiol. 2021;11:571416. doi: 10.3389/fphys.2020.571416 33510644 PMC7835928

[26] Crankson S, Pokhrel S, Anokye NK. Determinants of COVID-19-related length of hospital stays and long COVID in Ghana: a cross-sectional analysis. Int J Environ Res Public Health. 2022;19:527. doi: 10.3390/ijerph19010527 35010786 PMC8744866

[27] Mahmoodi Z, Bahrami G, Shahrestanaki E, et al. Clinical and socio-demographic variables associated with long COVID-19: a cross-sectional study. Clin Nurs Res. 2023;32:947–953. doi: 10.1177/10547738231177395 37264854 PMC10240294

[28] Lee JK, Son YJ. Gender differences in the impact of cognitive function on health literacy among older adults with heart failure. Int J Environ Res Public Health. 2018;15:2711. doi: 10.3390/ijerph15122711 30513761 PMC6313791

[29] Kazungu JS, Barasa EW. Examining levels, distribution and correlates of health insurance coverage in Kenya. Trop Med Int Health. 2017;22:1175–1185. doi: 10.1111/tmi.12912 28627085 PMC5599961

[30] Ilinca S, Di Giorgio L, Salari P, et al. Socio-economic inequality and inequity in use of health care services in Kenya: evidence from the fourth Kenya household health expenditure and utilization survey. Int J Equity Health. 2019;18:1–13. doi: 10.1186/s12939-019-1106-z 31849334 PMC6918604

[31] Ouma PN, Masai AN, Nyadera IN. Health coverage and what Kenya can learn from the COVID-19 pandemic. J Glob Health. 2020;10. doi: 10.7189/jogh.10.020362 PMC7565744 33110557

[32] Mohamed MS, Moulin TC, Schiöth HB. Sex differences in COVID-19: the role of androgens in disease severity and progression. Endocrine. 2021;71:3–8. doi: 10.1007/s12020-020-02536-6 33179220 PMC7657570

[33] Zeng F, Dai C, Cai P, et al. A comparison study of SARS‐CoV‐2 IgG antibody between male and female COVID‐19 patients: a possible reason underlying different outcome between sex. J Med Virol. 2020;92:2050–2054. doi: 10.1002/jmv.25989 32383183 PMC7267228

[34] Bajoulvand R, Hashemi S, Askari E, et al. Post-pandemic stress of COVID-19 among high-risk groups: a systematic review and meta-analysis. J Affect Disord. 2022;319:638–645. doi: 10.1016/j.jad.2022.09.053 36174783 PMC9512530

[35] Kamal M, Abo Omirah M, Hussein A, et al. Assessment and characterisation of post‐COVID‐19 manifestations. Int J Clin Pract. 2021;75:e13746. doi: 10.1111/ijcp.13746 32991035 PMC7536922

[36] Fitero A, Bungau SG, Tit DM, et al. Comorbidities, associated diseases, and risk assessment in COVID‐19—a systematic review. Int J Clin Pract. 2022;2022:1–24. doi: 10.1155/2022/1571826 PMC9640235 36406478

[37] Zhang X, Wang F, Shen Y, et al. Symptoms and health outcomes among survivors of COVID-19 infection 1 year after discharge from hospitals in Wuhan, China. JAMA Netw Open. 2021;4:e2127403–e2127403. doi: 10.1001/jamanetworkopen.2021.27403 34586367 PMC8482055

[38] Verveen A, Wynberg E, van Willigen HDG, et al. Severe fatigue in the first year following SARS-CoV-2 infection: a prospective cohort study. Vol. 9, Open forum infectious diseases. US: Oxford University Press; 2022. p. ofac127. 10.1093/ofid/ofac127 PMC8995073 35415196

[39] Heller O, Chun Y, Shapira S, et al. Prevalence of long-COVID among low-income and marginalized groups: evidence from Israel. Int J Public Health. 2022;67:1605086. doi: 10.3389/ijph.2022.1605086 36518871 PMC9742204

[40] van Kessel SAM, Olde Hartman TC, Lucassen L, et al. Post-acute and long-COVID-19 symptoms in patients with mild diseases: a systematic review. Fam Pract. 2022;39:159–167. doi: 10.1093/fampra/cmab076 34268556 PMC8414057

