# Supplementary material for: Recovery and long-term health outcomes of SARS-CoV-2 infection in a prospective cohort in an urban setting, Kenya
Source: Glob Health Action. 2025 May 30;18(1):2500795. doi: 10.1080/16549716.2025.2500795 (PMC12128128; doi:10.1080/16549716.2025.2500795)
Supplement: Supplementary files.docx [file ZGHA_A_2500795_SM6256.docx]

**Supplementary files**

**Supplementary Text 1:** Sample size calculation

**Supplementary Table 1a: Age and sex characteristics of populations excluded and included from study**

**Supplementary Table 1b:** Incidence proportion of 14 COVID-19-related symptoms according to study follow-up periods by SARS-CoV-2 infection severity

**Supplementary Table 2a:** Binomial GEE multivariable model on factors associated with fatigue over time

**Supplementary Table 2b:** Linear regression of factors associated with Quality of life (QoL) at 12 months after the first visit

**Supplementary Figure S1:** Flow chart of participant enrolment into the cohort study.

**Supplementary Figure S2:** Prevalence of COVID-19-related symptoms at the first study visit (x-axis).

**Supplementary Figure S3:** Overview of data collection in the Long COVID-19 study, Nairobi Kenya

**Supplementary Figure S4:** Transition between levels of self-reported severity of Long COVID over time among prospectively included participants, clinical severity group

**Supplementary Methods 1:** Illness Perception Questionnaire (IPQ-R)

**Supplementary Methods 2:** Fatigue Assessment Scale (FAS)

**Supplementary methods 3:** Test of the proportional-hazards assumptions after stcox using stcoxkm

**Supplementary Text 1:** Sample size calculation

Power calculations for various effect sizes were performed assuming that 50% of all participants diagnosed with COVID-19 would experience at least one symptom beyond three months from infection [1] and that one-third of these would be asymptomatic at diagnosis [2], a type 1 error of 𝛼=0.05, and a 95% confidence interval precision were used. A minimum sample of 129 symptomatic and 129 asymptomatic participants at diagnosis would be required to detect a difference of at least 20% (effect size, h=0.35) between the two groups (mild/moderate and severe/critical SARS-CoV-2 infection) with a power 80%, two-sided significance of 0.05 and using a Z- test. Assuming an estimated loss to follow-up of 30%, thus a total of 336 participants were to be recruited for follow-up.

**Supplementary Table 1a**: **Age and sex characteristics of populations excluded and included from study**

|  | Eligible | Refused to participate | Consented to participation | p-value |
| --- | --- | --- | --- | --- |
|  | N=750 | N =459**^†^** | N=291 |  |
| Sex (n, %) |  |  |  |  |
| Female | 420 (56.0) | 249 (54.2) | 172 (59.1) | 0.225 |
| Male | 330 (44.0) | 210 (45.8) | 119 (40.9) |  |
| Age, years (Median, IQR) | 38 (30-55) | 43 (31-61) | 34 (29-42) | 0.001 |
| **† Denotes the proportion of participants who refused to take part in the study after they were contacted and invited to take part in the study.** | | | | |

**Supplementary Table 1b: Incidence proportion of 14 COVID-19-related symptoms according to study follow-up periods by SARS-CoV-2 infection severity**

| **Follow-up period, months** | **first study visit*** | | | | | **3 months** | | | | | **6 months** | | | | |
| --- | --- | --- | --- | --- | --- | --- | --- | --- | --- | --- | --- | --- | --- | --- | --- |
| **Severity group** | **Overall** | **Mild/Moderate** | **Severe/ Critical** | **p-value** | **Overall** | | **Mild/Moderate** | **Severe/ Critical** | **p-value** | **Overall** | | **Mild/moderate** | **Severe/ Critical** | **p-value** |  |
|  |  |  |  |  |  |  |  |  |  |  |  |  |  |  |  |
| N→ | N=291 | N=249 | N=42 |  | N=251 | | N=218 | N=33 |  | N=229 | | N=198 | N=31 |  |  |
| Symptom ↓ |  |  |  |  |  |  |  |  |  |  |  |  |  |  |  |
| Pain | 107 (36.8) | 92 (37.0) | 15 (35.7) | 0.024 | 32 (12.7) | | 24 (11.0) | 8 (24.2) | 0.034 | 31 (13.5) | | 23 (11.6) | 8 (25.8) | 0.032 |  |
| Sore throat | 107 (36.8) | 93 (37.4) | 14 (33.3) | 0.620 | 40 (15.9) | | 36 (16.5) | 4 (12.1) | 0.52 | 26 (11.4) | | 21 (10.6) | 5 (16.1) | 0.367 |  |
| Nausea | 48 (16.5) | 35 (14.1) | 13 (31.0) | 0.006 | 21 (8.4) | | 19 (8.7) | 2 (6.1) | 0.608 | 5 (2.2) | | 4 (2.0) | 1 (3.2) | 0.669 |  |
| Breathlessness | 68 (23.4) | 51 (20.5) | 17 (40.5) | 0.005 | 28 (11.2) | | 23 (10.6) | 5 (15.2) | 0.434 | 17 (7.4) | | 9 (4.6) | 8 (25.8) | <0.001 |  |
| Weight loss | 41 (14.1) | 28 (11.2) | 13 (31.0) | 0.001 | 10 (4.0) | | 9 (4.1) | 1 (3.0) | 0.764 | 8 (3.5) | | 7 (3.5) | 1 (3.2) | 0.930 |  |
| Fatigue | 117 (40.2) | 93 (37.4) | 24 (57.1) | 0.016 | 43 (17.1) | | 33 (15.1) | 10 (20.3) | 0.031 | 26 (11.4) | | 20 (10.1) | 6 (19.4) | 0.131 |  |
| Stiff joints | 69 (23.7) | 49 (19.7) | 20 (47.6) | <0.001 | 22 (8.8) | | 17 (7.8) | 5 (15.2) | 0.164 | 19 (8.3) | | 13 (6.6) | 6 (19.4) | 0.016 |  |
| Sore eyes | 34 (11.7) | 29 (11.7) | 5 (11.9) | 0.962 | 21 (8.4) | | 18 (8.3) | 3 (9.1) | 0.872 | 10 (4.4) | | 6 (3.0) | 4 (12.9) | 0.012 |  |
| Wheeziness | 43 (14.8) | 32 (12.9) | 11 (26.2) | 0.024 | 18 (7.2) | | 15 (6.9) | 3 (9.1) | 0.647 | 8 (3.5) | | 4 (2.0) | 4 (12.9) | 0.002 |  |
| Headaches | 106 (36.4) | 89 (35.7) | 17 (40.5) | 0.555 | 37 (14.7) | | 30 (13.8) | 7 (21.2) | 0.261 | 26 (11.4) | | 24 (12.1) | 2 (6.5) | 0.355 |  |
| Upset Stomach | 36 (12.4) | 29 (11.7) | 7 (16.7) | 0.361 | 9 (3.6) | | 7 (3.2) | 2 (6.1) | 0.412 | 8 (3.5) | | 5 (2.5) | 3 (9.7) | 0.044 |  |
| Sleep difficulties | 62 (21.3) | 46 (18.5) | 16 (38.0) | 0.004 | 18 (7.2) | | 14 (6.4) | 4 (12.1) | 0.237 | 17 (7.4) | | 10 (5.1) | 7 (22.6) | 0.001 |  |
| Dizziness | 60 (20.6) | 48 (19.3) | 12 (28.6) | 0.168 | 19 (7.6) | | 15 (6.9) | 4 (12.1) | 0.289 | 17 (7.4) | | 11 (5.6) | 6 (19.4) | 0.006 |  |
| Loss of strength | 92 (31.6) | 71 (28.5) | 21 (50.0) | 0.006 | 27 (10.8) | | 21 (9.6) | 6 (18.2) | 0.14 | 11 (4.8) | | 8 (4.0) | 3 (9.7) | 0.172 |  |
| Overall symptoms | 187 (64.3) | 152 (61.0) | 35 (83.3) | 0.005 | 87 (34.7) | | 71 (32.6) | 16 (48.5) | 0.073 | 72 (31.7) | | 53 (26.8) | 19 (61.3) | 0.000 |  |
| ***** The median time between a positive PCR test and enrolment into the study was 99 days (IQR= 71-169) and 38.8% (n=113) out of 291 participants had less than 90 days between a positive PCR test and first visit. | | | | | | | | | | | | | | |  |

**Supplementary Table 1b continued**

| **Follow-up period, months** | **9 months** | | | | **12 months** | | | | |
| --- | --- | --- | --- | --- | --- | --- | --- | --- | --- |
| **Severity group** | **Overall** | **Mild/Moderate** | **Severe/ Critical** | **p-** | **Overall** | **Mild/Moderate** | **Severe/ Critical** | **p-value** |  |
|  |  |  |  | **value** |  |  |  |  |  |
| N→ | N=216 | N=188 | N=28 |  | N=219 | N=189 | N=30 |  |  |
| Symptom ↓ |  |  |  |  |  |  |  |  |  |
| Pain | 22 (10.2) | 17 (9.0) | 5 (17.9) | 0.15 | 34 (15.5) | 25 (13.2) | 10 (33.3) | 0.005 |  |
| Sore throat | 23 (10.6) | 17 (9.0) | 6 (21.4) | 0.035 | 30 (13.7) | 27 (14.3) | 3 (10.0) | 0.526 |  |
| Nausea | 6 (2.8) | 5 (2.7) | 1 (3.6) | 0.784 | 16 (7.3) | 12 (6.4) | 4 (13.3) | 0.172 |  |
| Breathlessness | 23 (10.6) | 15 (8.0) | 8 (28.6) | 0.001 | 26 (11.9) | 21 (11.1) | 5 (16.7) | 0.382 |  |
| Weight loss | 10 (4.6) | 8 (4.3) | 2 (7.1) | 0.498 | 9 (4.1) | 7 (3.7) | 2 (6.7) | 0.448 |  |
| Fatigue | 24 (11.1) | 21 (11.2) | 3 (10.7) | 0.943 | 37 (16.9) | 29 (15.3) | 8 (26.7) | 0.124 |  |
| Stiff joints | 13 (6.0) | 9 (4.8) | 4 (14.3) | 0.049 | 20 (9.1) | 17 (9.0) | 3 (10.0) | 0.859 |  |
| Sore eyes | 9 (4.2) | 8 (4.3) | 1 (3.6) | 0.866 | 7 (3.2) | 5 (2.7) | 2 (6.7) | 0.245 |  |
| Wheeziness | 8 (3.7) | 3 (1.6) | 5 (17.9) | <0.001 | 5 (2.3) | 3 (1.6) | 2 (6.7) | 0.084 |  |
| Headaches | 25 (11.6) | 23 (12.2) | 2 (7.1) | 0.432 | 29 (13.2) | 26 (13.8) | 3 (10.0) | 0.573 |  |
| Upset Stomach | 2 (0.9) | 2 (1.1) | 0 (0.0) | 0.583 | 11 (5.0) | 8 (4.2) | 3 (10.0) | 0.176 |  |
| Sleep difficulties | 14 (6.5) | 11 (5.9) | 3 (10.7) | 0.329 | 18 (8.2) | 12 (6.3) | 6 (20.0) | 0.011 |  |
| Dizziness | 14 (6.5) | 9 (4.8) | 5 (17.9) | 0.009 | 20 (9.1) | 14 (7.4) | 6 (20.0) | 0.026 |  |
| Loss of strength | 16 (7.4) | 10 (5.3) | 6 (21.4) | 0.002 | 22 (10.0) | 16 (8.5) | 6 (20.0) | 0.051 |  |
| Overall symptoms | 66 (30.6) | 51 (27.1) | 15 (53.6) | 0.005 | 68 (31.1) | 53 (28.0) | 15 (50.0) | 0.016 |  |

**Supplementary Table 2a: Binomial GEE multivariable model on factors associated with fatigue over time**

| **Fatigue** | | | |
| --- | --- | --- | --- |
| **Factors (Selected prior)** | **n (%)** | **aOR (95% CI)** | **p-value** |
| **Sex** |  |  | 0.006 |
| Male | 172 (59.1) | Ref |  |
| Female | 119 (40.9) | 1.781 (1.128 to 2.811) |  |
| **Age group** |  |  | 0.735 |
| <40 | 198 (68.0) | Ref |  |
| 40-64 | 79 (27.1) | 0.999 (0.596 to 1.674) |  |
| ≥65 | 14 (4.8) | 0.843 (0.266 to 2.672) |  |
| **Education level** |  |  | 0.515 |
| Primary | 27 (9.3) | Ref |  |
| Secondary | 48 (16.5) | 0.677 (0.313 to 1.464) |  |
| Tertiary | 216 (74.2) | 0.699 (0.354 to 1.381) |  |
| **Socioeconomic status** |  |  | 0.001 |
| Low class | 32(11.0) | Ref |  |
| Middle class | 158 (54.3) | 0.977 (0.678 to 1.408) |  |
| Upper class | 68 (23.4) | 0.507 (0.316 to 0.813) |  |
| Missing | 33 (11.3 |  |  |
| **Employment** |  |  | 0.047 |
| No | 41 (14.1) | Ref |  |
| Yes | 250 (85.9) | 0.518 (0.273 to 0.981) |  |
| **BMI, Kg/M^2^** |  |  | 0.767 |
| Normal Weight | 126 (43.3) | Ref |  |
| Overweight | 95 (32.6) | 1.159 (0.686 to 1.958) |  |
| Obese | 70 (24.1) | 0.9 (0.510 to 1.586) |  |
| **Comorbidity** |  |  | 0.743 |
| None | 219 (75.3) | Ref |  |
| One or more | 72 (24.7) | 1.1 (0.623 to 1.94) |  |
| **Covid-19 Clinical severity ^b^** |  |  | 0.005 |
| Severe/critical | 42 (14.4) | Ref |  |
| Mild/Moderate | 249 (85.6) | 0.39 (0.199 to 0.764) |  |
| **Vaccination status** |  |  | 0.566 |
| Vaccinated | 255 (87.6) | Ref |  |
| Unvaccinated | 36 (14.4) | 1.225 (0.651 to 2.302) |  |
| **Days^c^** |  | 0.999 (0.998 to 1) | 0.010 |
| aOR: Adjusted Odds ratio, CI: Confidence Interval. Fatigue was measured using fatigue assessment scale (FAS) and Fatigue: defined as no fatigue (FAS score <22) and Fatigue (FAS score score ≥ 22). Clinical severity groups are defined as follows: Mild/moderate SARS-CoV-2 infection was defined as participants who reported not having had hospital admissions and oxygen therapy due to COVID-19 as well as home-based care and oxygen therapy and severe otherwise. Severe/critical SARS-CoV-2 infection was defined as participants reported having had hospital admission and/or needed oxygen therapy due to COVID-19.  ^c^Time since positive PCR | | | |

**Supplementary Table 2b: Linear regression of factors associated with Quality of life (QoL) at 12 months after the first visit**

| **Quality of life at 12 months after first visit** | | | | | |  |
| --- | --- | --- | --- | --- | --- | --- |
|  |  | **Univariate Model** | | **Multivariate Model** | |  |
| **Factors** | **n (%)** | **β coef. (95% CI)** | **P-value** | **β coef. (95% CI)** | **P-value** | |
| Sex |  |  | 0.006 |  | 0.051 | |
| Male | 84 (38.4) | Ref |  | Ref |  | |
| Female | 135 (61.6) | -5.045 (-8.601 - -1.49) |  | -2.166 (-5.112 - 0.779) |  | |
| Age group (years) |  |  | <0.001 | | 0.029 | |
| <40 | 150 (68.5) | Ref |  | Ref |  | |
| 40-65 | 59 (26.9) | -5.745 (-10.091 - -1.399) |  | -2.116 (-5.945 - 1.712) |  | |
| >65 | 10 (4.6) | -23.84 (-36.803 - -10.87) |  | -11.62 (-22.45 - -0.793) |  | |
| Education |  |  | <0.001 | | 0.650 | |
| Primary | 17 (7.8) | Ref |  | Ref |  | |
| Secondary | 36 (16.4) | 13.254 (3.52 - 22.988) |  | 5.286 (-0.822 - 11.394) |  | |
| Tertiary | 166 (75.8) | 16.021 (7.241 - 24.802) |  | 11.165 (4.764 - 17.567) |  | |
| Socioeconomic status | |  | <0.001 |  | <0.001 | |
| Low class | 22 (10.0) | Ref |  |  |  | |
| Middle class | 127 (58.0) | 8.11 (0.512 - 15.707) |  |  |  | |
| Upper class | 70 (32.0) | 16.4 (8.869 - 23.931) |  |  |  | |
| BMI (KG/M2) |  |  | 0.338 |  | 0.732 | |
| Normal | 87 (39.7) | Ref |  | Ref |  | |
| Overweight | 79 (36.1) | 1.564 (-2.341 - 5.469) |  | 0.417 (-2.899 - 3.733) |  | |
| Obesity | 53 (24.2) | -3.035 (-8.282 - 2.212) |  | 1.019 (-2.666 - 4.705) |  | |
| Comorbidity |  |  | 0.001 |  | 0.906 | |
| None | 165 (75.3) | Ref |  | Ref |  | |
| One or more | 54 (24.7) | -8.551 (-13.482 - -3.62) |  | 0.378 (-4.014 - 4.769) |  | |
| SARS-CoV-2 severity |  |  | <0.001 | | 0.005 | |
| Severe/critical | 30 (13.7) | Ref |  | Ref |  | |
| Mild/Moderate | 189 (86.3) | 16.688 (9.27 - 24.105) |  | 10.508 (3.217 - 17.8) |  | |
| Symptoms | 68 (31.0) | -2.295 (-3.315 - -1.276) | <0.001 | -1.705 (-2.595 - -0.816) | <0.001 | |
| Days^c^ | NA | NA | NA | -0.013 (-0.03 -1 0.005) | 0.161 | |
| CI: Confidence Interval. NA: Not Applicable, QoL was measured using the SF-36 questionnaire and Quality of life (QoL): was measured using SF-36 scores ranging from 0 (worst) to 100 (best). Clinical severity groups are defined as follows: Mild/moderate SARS-CoV-2 infection was defined as participants who reported not having had hospital admissions and oxygen therapy due to COVID-19 as well as home-based care and oxygen therapy and severe otherwise. Severe/critical SARS-CoV-2 infection was defined as participants reported having had hospital admission and/or needed oxygen therapy due to COVID-19.  The median time between a positive PCR test and enrolment into the study was 99 days (IQR= 71-169) and 38.8% (n=113) out of 291 participants had less than 90 days between a positive PCR test and first visit. | | | | | |  |

**Identification**

**Screening**

**Eligibility**

**Included**

2145 patients screened for inclusion

1835 patients tested for COVID-19 at KUTRRH

300 patients tested for COVID-19 at KEMRI

10 walk-in patients

Patients with SARS-CoV-2 PCR test from December 2020 to July 2022 were selected

1395 patients excluded

- PCR test negative
- Clinically diagnosed
- Live outside Nairobi Metropolitan area

750 patients were invited to participate

459 patients refused to participate

291 patients included in the study

**Supplementary Figure S1:** Flow chart of participant enrolment into the cohort study.

**Supplementary Figure S2:** Prevalence of COVID-19-related symptoms at the first study visit (x-axis). The median time between a positive PCR test and enrolment into the study was 99 days (IQR= 71-169) and 38.8% (n=113) out of 291 participants had less than 90 days between a positive PCR test and first visit.


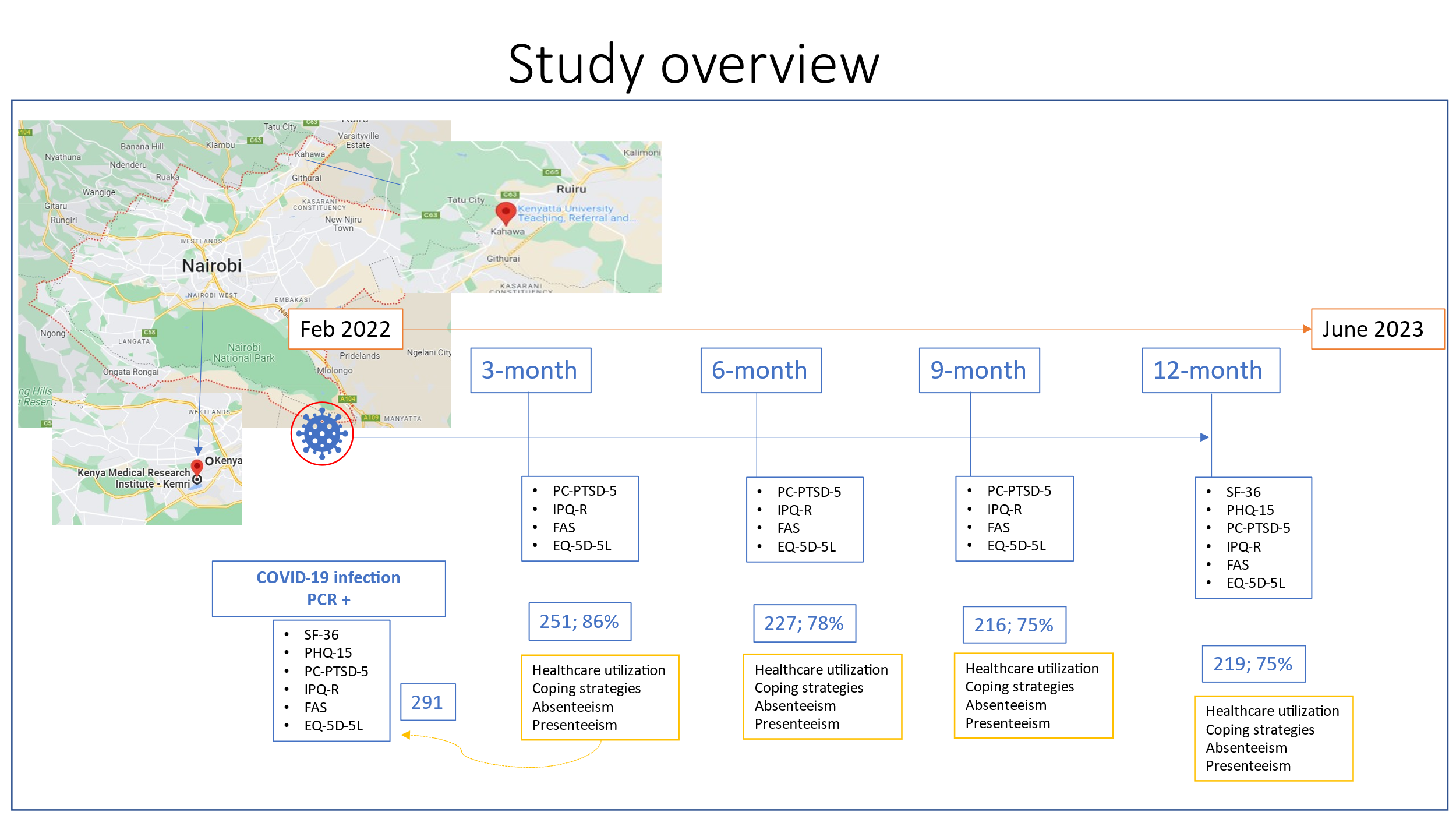
Questionnaires: 36-items short form health survey (SF-36); Patient Health Questionnaire-15 (PHQ-15); Primary care PTSD screen for DSM-5 (PC-PTSD-5); Illness Perception Questionnaire (IPQ-R); Fatigue Assessment Scale (FAS); EuroQol 5 Dimension 5- Level (EQ-5D-5L).

**Supplementary Figure S3: Overview of data collection in the Long COVID-19 study, Nairobi Kenya**

**Supplementary Figure S4. Transition between levels of self-reported severity of Long COVID over time among prospectively included participants, clinical severity group**

The vertical bars represent the number of participants for each severity level of the COVID-19-related symptom. Initial COVID-19 clinical severity groups are defined as follows: Mild/moderate SARS-CoV-2 infection was defined as participants who reported not having had hospital admissions and oxygen therapy due to COVID-19 as well as home-based care and oxygen therapy and severe otherwise. Severe/critical SARS-CoV-2 infection was defined as participants reported having had hospital admission and/or needed oxygen therapy due to COVID-19. The size of the streams indicates the number of study participants who moved from one severity level to another; transitioning from any severity level to “No” represents recovery from that symptom.


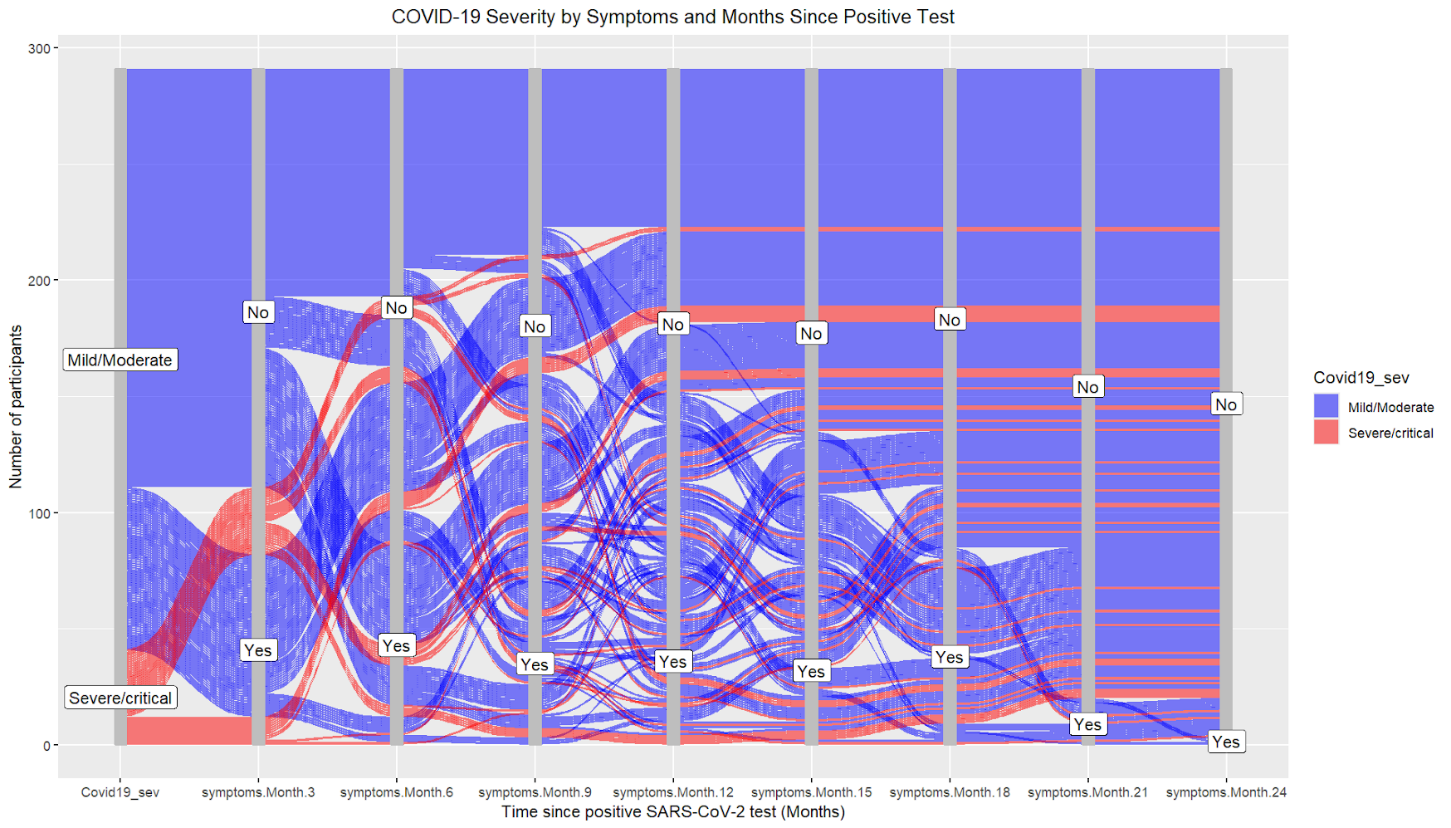


**Supplementary methods**

**Supplementary Methods 1: Illness Perception Questionnaire (IPQ-R)**

Your views about your illness

Listed below are a number of symptoms that you may or may not have experienced since your lung disease. Please indicate by circling Yes or No, whether you have experienced any of these symptoms since your COVID-19 disease or last follow-up visit, and whether you believe that these symptoms are related to your COVID-19 disease.

**I have experienced this symptom *since my COVID-19 disease/the last follow up visit***

**This symptom is *related to my COVID-19 disease***

Pain Yes No ________________ Yes No

Sore Throat Yes No ________________ Yes No

Nausea Yes No ________________ Yes No

Breathlessness Yes No ________________ Yes No

Weight Loss Yes No ________________ Yes No

Weight Gain Yes No ________________ Yes No

Fatigue Yes No ________________ Yes No

Stiff Joints Yes No ________________ Yes No

Sore Eyes Yes No ________________ Yes No

Wheeziness Yes No ________________ Yes No

Headaches Yes No ________________ Yes No

Upset Stomach Yes No ________________ Yes No

Sleep Difficulties Yes No ________________ Yes No

Dizziness Yes No ________________ Yes No

Loss of Strength Yes No ________________ Yes No

**Supplementary Methods 2: Fatigue Assessment Scale (FAS)**

The following 10 statements refer to how you usually feel. Per statement you can choose one out of five answer categories, varying from Never to Always. *1=Never; 2= Sometimes (about monthly or less); 3=Regularly (about a few times a month); 4= Often (about weekly); 5= Always (about every day).*

|  |  | Never | Sometimes | Regularly | Often | Always |
| --- | --- | --- | --- | --- | --- | --- |
| 1 | I am bothered by fatigue | 1 | 2 | 3 | 4 | 5 |
| 2 | I get tired very quickly | 1 | 2 | 3 | 4 | 5 |
| 3 | I don’t do much during the day | 1 | 2 | 3 | 4 | 5 |
| 4 | I have enough energy for everyday life | 1 | 2 | 3 | 4 | 5 |
| 5 | Physically, I feel exhausted | 1 | 2 | 3 | 4 | 5 |
| 6 | I have problems to start things | 1 | 2 | 3 | 4 | 5 |
| 7 | I have problems to think clearly | 1 | 2 | 3 | 4 | 5 |
| 8 | I feel no desire to do anything | 1 | 2 | 3 | 4 | 5 |
| 9 | Mentally, I feel exhausted | 1 | 2 | 3 | 4 | 5 |
| 10 | When I am doing something, I can concentrate quite well | 1 | 2 | 3 | 4 | 5 |

**Supplementary methods 3: Test of the proportional-hazards assumptions after stcox using stcoxkm**

To verify the proportional hazards assumption in our Cox proportional hazards model, we used the stcoxkm command. This method compares the observed survival curves with the predicted survival curves under proportional hazards. Our analysis indicates that the proportional hazards assumption for COVID-19-related severity has not been violated where the observed and predicted values are close together. This suggests that the relationship between the covariates and the hazard of COVID-19-related severity remains consistent over time, validating the use of the Cox model in the study.

**References**

1. Lopez-Leon S, Wegman-Ostrosky T, Perelman C, et al. More than 50 long-term effects of COVID-19: a systematic review and meta-analysis. Sci Rep **2021**; 11:1–12.

2. Huang Y, Pinto MD, Borelli JL, et al. COVID symptoms, symptom clusters, and predictors for becoming a Long-Hauler looking for clarity in the haze of the pandemic. Clin Nurs Res **2022**; 31:1390–1398.
